# Pattern Recognition Receptors: Significance of Expression in the Liver

**DOI:** 10.1007/s00005-020-00595-1

**Published:** 2020-09-17

**Authors:** Jan Żeromski, Agata Kierepa, Bartosz Brzezicha, Arleta Kowala-Piaskowska, Iwona Mozer-Lisewska

**Affiliations:** 1 Chair of Pathomorphology and Clinical Immunology, Karol Marcinkowski University of Medical Sciences, Poznan, Poland; 2 Chair and Department of Infectious Diseases, Hepatology and Acquired Immunodeficiencies, Karol Marcinkowski University of Medical Sciences, Poznan, Poland

**Keywords:** PRRs, TLRs, Liver, Hepatitis, Hepatic fibrosis, Hepatocarcinogenesis

## Abstract

Pattern recognition receptors (PRRs) are a pivotal part of the immune system. They are distributed in almost every site of higher organisms, able to recognize foreign pathogens or unwanted remnants of metabolism and mount innate immune response. Moreover, PRRs create bridging signaling to initiate adaptive immunity. The liver being the largest organ of the body, exposed to myriads of foreign substances often being immunogenic, is well equipped with PRRs. They act as sentinels of the organ, both in health and disease. In viral hepatitis C at least two of them, RIG-1 and TLR3 sense HCV, induce protective interferon production and create proinflammatory status. The hepatitis B virus is apparently invisible to PRRs, which has recently been denied. Besides, they are active in the course of infection. In liver injury and hepatic fibrogenesis Toll-like receptors (TLRs), predominantly TLR4, TLR3 and TLR9 are associated with gut microflora-related products and DNA from dying hepatocytes, lead to the activation of hepatic stellate cells. The latter initiate production of fibrillar collagens, the main agents forming hepatic fibrosis. Tumor cells of primary liver cancer also express PRRs, mainly TLRs. In concert with non-resolving liver inflammation, they are considered pivotal factors leading to carcinogenesis.

## Introduction

Pattern recognition receptors (PRRs) are evolutionally very old, germ line encoded receptors expressed on cell membranes and also in the cytoplasm of several cells but predominantly on those of the immune system. They are present in most of *Eukariota* but absent in *Prokariota*, such as unicellular bacteria, protozoa, viruses and fungi (Lemaitre et al. 1996). PRRs are considered to be pivotal members of innate (non-specific) immunity and they form a bridging pathway between innate and adaptive immune responses (Motta et al. 2015; Schenten and Medzhitov 2011). PRRs are grouped into six families, subdivided in sensors, each able to “see” a given molecular compound. Sensors recognize so called molecular patterns of two types. The first, named PAMPs (pathogen-associated molecular patterns) sense various substances pivotal for the growth and proliferation of unicellular organisms. The second, so-called DAMPs (danger-associated molecular patterns) recognize unwanted or noxious molecules of higher multicellular organisms such as uric acid and occasional chemical compounds, for example silica. The earliest discovered and described PRRs were Toll-like receptors (TLRs) in the fruit fly (Lemaitre et al. 1996) as antifungal agents. There are at least ten TLR items in humans, but more have been found in various animals, such as mice—13. Recognition of a particular TLR by a given compound results in downstream activation of different adaptor proteins, several cytoplasmic enzymes and other molecules leading to the maturation and assembly of transcription factors such as nuclear factor (NF)-κB, activator protein (AP)-1, interferon regulating factors (IRFs) and others. The latter enter the cell nucleus and initiate transcription of several genes encoding proinflammatory cytokines, cell adhesion molecules, MHC antigens, etc. Apart from the activation of cell metabolism, several adverse phenomena may take place in a cell. These include cell apoptosis, autophagy or even pyroptosis (cell death due to excessive inflammation) (Akira et al. 2006; Banerjee and Gerondakis 2007; Medzhitov 2013). The expression of TLRs has been demonstrated as transmembrane structures or cytoplasmic ones in cells of several human internal organs, including the liver. In the latter, practically all cell types have been found to express both RNA and protein of most known PRRs. As expected, Kupffer cells, due to their broad cell-intrinsic innate functions, have the highest expression of PRRs. Others, such as hepatocytes, hepatic stellate cells (HSC) or liver sinusoidal endothelial cells are also positive but to a lesser degree. Cells express most frequently not only TLRs, but also RIG-1-like ones, DNA cytosolic sensors as well as NOD-like receptors (NLRs) (Alegre et al. 2017; Faure-Dupuy et al. 2018; Kiziltas 2016).

Apart from the significance of PRRs in various pathological conditions of the liver described below, there is a growing interest in the role of these receptors in a normal organ. For example, Lang et al. (2006) demonstrated on the example of animal experiments (mice) that the immunoprivileged status of the liver is under control of TLR3 signaling. TLR3 activation-induced interferon (IFN)-α and tumor necrosis factor (TNF)-α release, which subsequently resulted in expression of hepatic CXCL9 chemokine. The latter enhanced CD8^+^T cell infiltration and liver injury in mice.

## Viral Hepatitis

### Hepatitis C

**Fig. 1:**
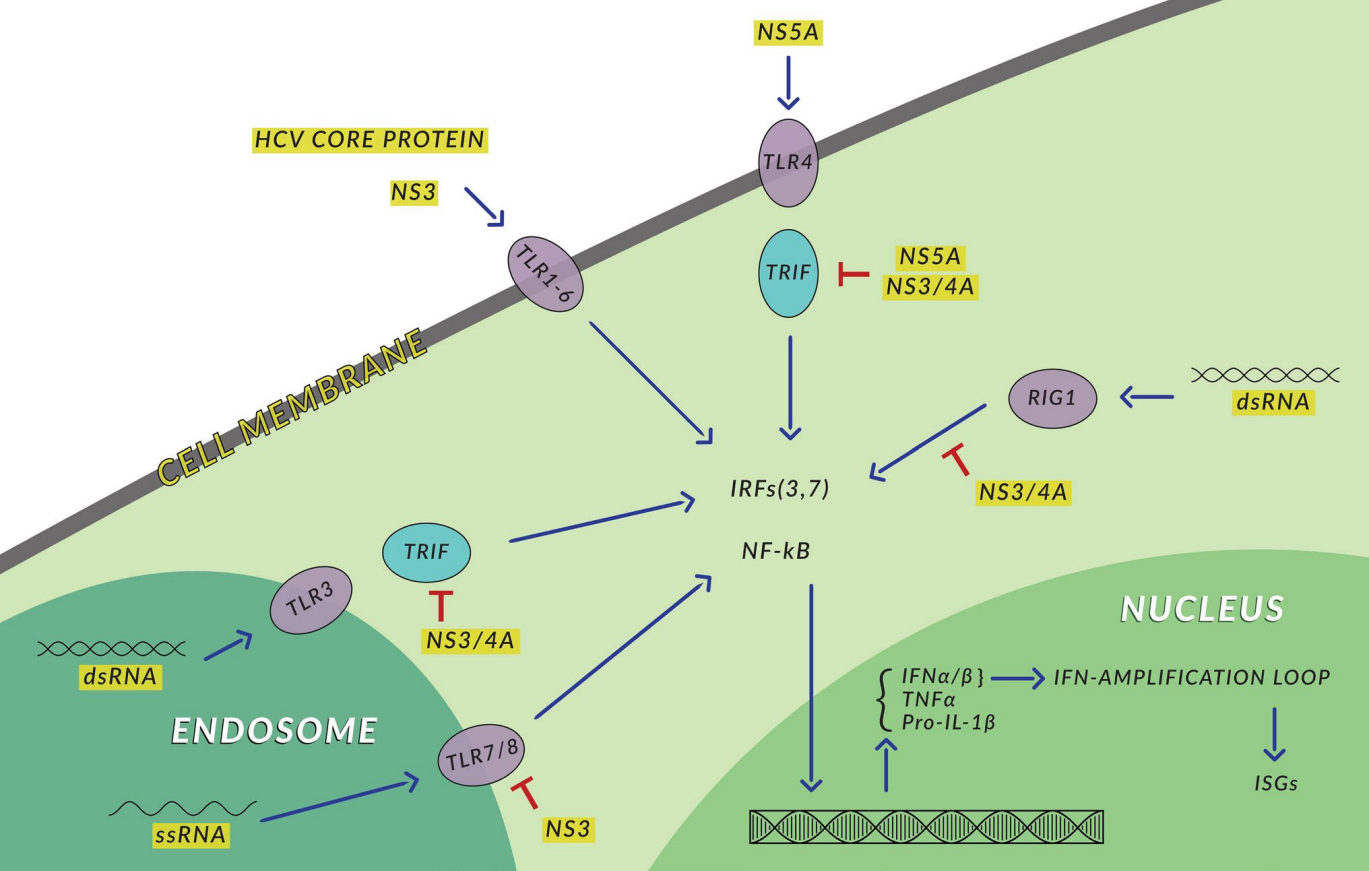
Hepatitis C. Inhibition of protective immune responses in hepatocyte by viral proteins. *dsRNA* double-stranded RNA, *ssRNA* single stranded RNA. Details in the text

Expression of PRRs in hepatitis B and C was noticed by several authors, including ourselves (Mozer-Lisewska et al. 2010). Two PRRs RIG-1 and TLR3 are considered crucial to establish antiviral conditions in an organism infected with hepatitis C virus (HCV). They both sense double-stranded RNA, essential in the HCV replication cycle, activate transcription factors IRF3 and IRF7, which results in the synthesis of type IFNs anti-HCV agents. HCV reduces TRIF, a critical TLR3 adaptor, which hampers the IFN antiviral effect (Wang et al. 2009). Imran et al. (2012) searched the data from PubMed and Google Scholar concerning the effect of the HCV proteins on PRRs in the liver. They found that some of these proteins exert negative impact on PRRs pathways, helping to evade host immune response. For example, core monomeric protein stimulates the TLR2 pathway, helping the virus to evade host innate immunity. NS3/4A damage TLR3 and RIG-1 pathways. NS5A decrease expression NKG2D on natural killer (NK) cells via the TLR4 pathway, which results in the impairment of the functioning of these cells. The HCN NS3/4A protease was also revealed to cause specific proteolysis of adaptor protein TRIF linking TLR3, which impairs the signaling of the TLR3 pathway, promoting persistent infections (Li et al. 2005). These data unequivocally show direct interactions between the HCV proteins and the PRRs one (Fig. 1).

Interesting data have been demonstrated in relation to the interaction between plasmacytoid dendritic cells (pDCs) and C virus-infected hepatocytes. It was shown that type I IFN production is only made via TLR7 signaling, when both cell types are in direct contact but pDCs do not become infected, in spite of apparent infusion of viral RNA. Moreover, IFN production requires the concordance of cell species (human/human) and highly differentiated hepatocytes. It was concluded by the authors that this phenomenon constitutes a novel cell–cell viral RNA transfer process, which might have an important role in the course and treatment of HCV-infected patients (Takahashi et al. 2010).

In the chronic stage of this disease at least six TLRs mRNA levels assessed by real time PCR were substantially upregulated in monocytes and T cells as compared to controls. Upregulation was somewhat selective because TLR4 was only increased in T cells, while TLR5 exclusively in monocytes. One explanation of this phenomenon provided by the authors was the possible innate recognition of pathogens and an attempt to mount protective immune response by HCV-bearing individuals (Dolganiuc et al. 2006). Previously, these authors indicated that the HCV core and the NS3 proteins triggered in monocytes several proinflammatory agents, such as interleukin (IL)-1 receptor-associated kinase, extracellularly regulated and c-jun N-terminal kinases and AP-1 via TLR2 in mice. However, it was not the case in the TLR2 knockout animals (Dolganiuc et al. 2004). On the other hand, Motavaf et al. (2014) by means of sensitive DNA-based technology found significantly lower expression of TLR3 and TLR7 in peripheral blood mononuclear cell (PBMC) of 25 patients with chronic HCV infection as compared to healthy controls. It was of interest, because both mentioned TLR members recognize viral RNA in PBMC of HCV^+^ patients. The low expression of those TLRs may reflect the way of viral evasion of host innate immunity.

Homozygous TLR genotype association appears to be protective in chronic HCV. Mosaad et al. (2019) showed that the heterozygous CT genotype of TLR3rs 3775290 appeared to be a susceptibility risk factor of chronic HCV infection, while the homozygous CC and of TLR7rs 179008, TLR9rs 352140 single nucleotide polymorphism (SNPs), including the combined genotype (CC-AT-GA) were protective. Earlier, another group of authors (Hamdy et al. 2018) found that the TLR3rs 3775290, “CC” genotype was associated with chronic HCV infection, this SNP, however, did not correlate with the HCV RNA load of the chronic subjects. These studies indicate the potential value of genetic exploration of TLRs in this disease, in spite of evident discrepancies between individual reports.

Another group of Egyptian investigators (Mohamed et al. 2017) demonstrated links between the TLR4 expression and liver fibrosis in chronic HCV patients. In the cohort of 50 patients, fibrosis progression was positively associated with TLR4 expression. The latter was also positively correlated with several other parameters such as age, fibrosis stage, HCV RNA, serum transaminases, total bilirubin and prothrombin time but negatively with platelet count and serum albumin. The authors suggested that anti-TLR4 antibodies might limit the progression of fibrosis.

Some TLRs agonists were tested as potential adjuncts of anti-HCV therapy. As early as in 2009, Libri et al. (2009) found that out of four prototypic synthetic agonists of TLR7 or TLR9, only class C CpG oligodeoxynucleotide (CpG-ODN), TLR9 agonist was able to induce a robust IFN-α secretion by pDCs in patients with chronic HCV infection. Other proinflammatory cytokines production by infected pDC in response to all four agonists did not differ from the uninfected ones. It suggested a selective defect in IFN-α production by pDC from chronic HCV patients. In another study, Yuki et al. (2010) showed that low TLR3 expression was associated with good response to the treatment by peginterferon plus ribavirin in genotype 1 patients.

The impact of the direct acting antivirals (DAA) therapy on regulatory T cells (Tregs) and T helper (Th)17 cell response to TLR2 agonist Pam3Csk4 in treated HCV patients were researched (Wei et al. 2018). The treatment resulted in the downregulated responsiveness of the abovementioned cells to TLR2 stimulation, which was interpreted as a novel mechanism of DAA-induced immunoregulation.

### Hepatitis B

The situation is distinct in the case of hepatitis B virus (HBV). The virus is apparently “invisible” to the innate immune system, which was proven in both animals and human (Suslov et al. 2018). HBV-infected liver cells had the same expression of IFN or IFN stimulating genes as cells from non-infected controls. These stealth properties of the HBV are apparently not associated with the infected liver, because the IFN-α response could be induced by other means, such as the Sendai virus infection. The authors concluded that HBV is “invisible” to PRRs. It has, however, been recently shown that the HBV is able to induce innate immune responses in primary human hepatocytes. Following exposure to the HBV particles, hepatocytes demonstrated nuclear translocation of NF-κB, the secretion of proinflammatory cytokines, such as IL-1β, IL-6, TNF. The HBV-induced gene expression profile was comparable to that of a TLR2 ligand (Pam3Cys) but distinct from those known as viral sensors, TLR3 or TLR7/9. These findings contrast with the abovementioned stealth properties of HBV (Zhang et al. 2020). On the other hand, Guo et al. (2009) studied the effect of the PRRs adaptors (TRIF, RIG-1/MDA5, IPS-1) on HBV^+^ co-transfected cells (HepG2 and Huh7). It was shown that each of the three adaptors markedly reduced the levels of HBV mRNA and DNA in both cell types. Studies on TLRs polymorphisms in HBV infection also did not exclude the role of interactions of viral molecules with TLRs in disease. The TT genotype of the missense variant TLR5 was found to be significantly more frequent in the control group than in HBV patients, indicating the protective effect of this abnormality against infection (Katrinli et al. 2018). Similar findings were also noticed for the TLR3 gene (EzzEI-Din et al. 2017). The authors compared six SNPs in chronic HBV carriers and HBV-immunized individuals. The latter showed high frequencies of selected haplotypes (GCTCCA and CCA) as compared to chronic HBV groups. Askari et al. (2016) and Al-Anazi et al. (2019) studied nucleotide oligomerization domain 2 (NOD2) of NLRs and myeloid differentiation protein 2 (MD2), co-receptor for TLR4, polymorphisms in HBV-infected patients. They found that homozygosity of selected haplotypes of both, NOD2 gene and MD2-gene were significantly associated with HBV infection. (Boni et al. 2018) studied the TLR7 agonist (GS 9620 nucleoside analogue) in 28 HBV patients as the therapy of HBV infection. They found greater responses to HBV and increased activation of T cells, manifested by higher levels of produced cytokines than in the treatment-naive group. NK cell activation and function were also increased. A 12-week therapy did not have major impact on serum hepatitis B surface antigen levels and did not significantly increase T-cell and NK-cell responses. The authors concluded that GS 9620 might be possibly applied in the therapy of HBV infection to increase specific cell-mediated immune responses.

## Liver Fibrosis

**Fig. 2:**
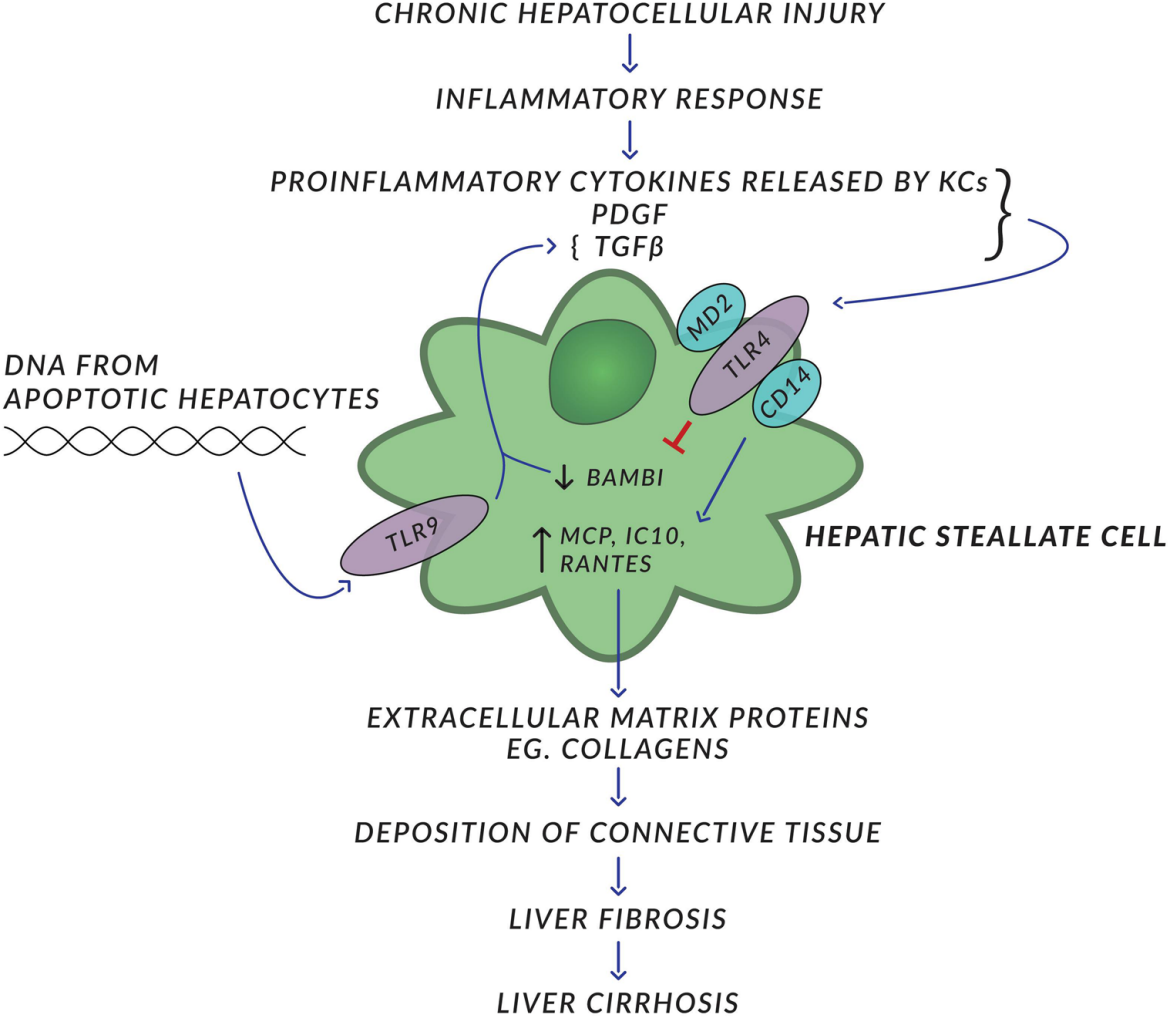
Factors leading to hepatic fibrosis. *PDGF* platelet-derived growth factor, *KC* Kupffer cell. Details in the text

Liver fibrosis is a consequence of most hepatic pathologic disorders such as hepatitis, alcoholic liver disease, or fatty liver. Chronic hepatocellular injury followed by inflammatory response results in the production of several proinflammatory and/or immunosuppressive cytokines and accumulation of the variety of inflammatory cells. This inflammatory microenvironment activates HSC, which initiate the production of several extracellular matrix proteins, such as various subtypes of collagen. This leads to the deposition of connective tissue among hepatocytes, which leads to liver fibrosis and eventually to its more severe form, namely cirrhosis. Fibrogenic stimuli acting on HSC include TGF- β, platelet-derived growth factor, proinflammatory cytokines produced by Kupffer cells and others (Friedman 2008). Human HSC express TLR4 and its co-receptors MD2 and CD14 (Paik et al. 2003). TLR4 was found to upregulate several chemokines, such as MCP, RANTES, IP10 and to inhibit Bambi-type I TGF-β receptor but to be suppressant of TGF-β, activin and bone morphogenetic protein. Inhibition of Bambi by TLR4 enhances TGF- β signaling in HSC and thus has promoting effect on the augmentation of liver fibrosis (Guo and Friedman 2010). It is suggested that high levels of Bambi expression inhibit TGF-β signaling in HSC of normal livers (Aoyama et al. 2010) Apart from TLR4, HSC express also TLR2, TLR3 and TLR9. All upregulate HSC and TLR9 does it under the influence of host-derived denatured DNA from apoptotic hepatocytes (Fig. 2). Thus, TLR9 expressed on HSCs recognizing CpG-rich DNA seems to be another profibrotic agent. This leads to the differentiation of HSC and inhibition of their chemotaxis (Watanabe et al. 2007). Kupffer cells also participate in the formation of liver fibrosis through the activation of TLRs by lipopolysaccharides. It results in the production of several cytokines, including fibrogenic ones. The most significant of them all appears to be transforming growth factor (TGF)-β, binding receptor (TGF-β R1) on HSCs, which leads to the production and release of collagen fibers (Seki et al. 2007). Recently, Chinese and American authors have reported that mice with deleted TLR4 have been protected from chronic alcohol-induced liver disease and fatty liver (Jia et al. 2018). Stimulated NK cells are cytotoxic for activated HSCs via IFN type 1. TLR3 ligand poly:IC and IFN-γ apparently reduce liver fibrosis through TRAIL expression in NK cells (Radaeva et al. 2006). Cirrhotic individuals showed increased bacterial DNA in their plasma. Moreover, as mentioned earlier, denatured host origin DNA from dying cells stimulates HSCs via TLR9, which enhances liver fibrosis (Gabele et al. 2008).

Another group of investigators (Hartmann et al. 2012) working on genetically modified mouse models showed the crucial role of TLR2, bacteria and bacterial products translocation across the intestinal epithelial lining in concert with TNF receptor 2 signaling for the contribution to liver injury and subsequently to liver fibrosis. TNFR2 is produced by monocytes via TLR2 signaling. These data underline the role of TLRs involved in the initiation of liver fibrosis.

Apart from TLRs, another family of PRRs, namely NLR participate in liver fibrosis. Some of them are able to form bioactive protein complexes called inflammasomes, releasing proinflammatory cytokines IL-1β and IL-18, which initiate the activation of HSCs in chronic inflammatory liver diseases (Alegre et al. 2017; Wang et al. 2019). It has recently been reported that a selective inflammasome (NLRP3) inhibitor—MCC950 is able to alleviate liver fibrosis in mice (Ou et al. 2019).

## PRRs and Liver Carcinogenesis

There are several reports, predominantly by Chinese authors but also ourselves (Żeromski et al. 2019) about the frequency of expression and significance of TLRs in hepatocellular carcinoma (HCC). For example, Sun et al. (2016) showed that about 53 and 85% of patients bearing liver cancer express TLR3 and TLR9, respectively. Tanaka et al. (2010) showed that TLR9 agonist CpG-ODN promoted HCC cell proliferation but reduced cytotoxicity of anticancer drug adriamycin. Moreover, TLR agonists closely regulated genes and transcription factors involved in tumorigenesis and progression of HCC as shown by means of gene chip microassay analysis. Non-resolving inflammation is considered an important causative factor of liver cancer (Yu et al. 2018). In mice deleted for TGF β-activated kinase 1 (Tak1Hep) known to develop inflammation and HCC, TLR4 and TLR9-MyD88 were found to be the driving forces of progression to cancer (Song et al. 2018). Zhou et al. (2019) noticed that high expression of TLR2 and TLR4 on PBMC was associated with intestinal overgrowth promoted the development of HCC. Another group (Hou et al. 2017) reported that the expression of hepatitis B core antigen in concert with the presence of TLR4 promoted invasion of the established HCC cell line. On the other hand, TLR3 expression on HCC cells seems to trigger cell apoptosis. Thus, Bonnin et al. (2019) concluded, based on experimental work, that TLR3 downregulation appears to be a novel escape way from apoptosis in carcinogenesis of liver. Yu et al. (2010) and later Yu et al. (2018) in a review article came to the conclusion that the activation of PRRs by PAMPs or unwanted host products from dying liver cells leading to unresolving inflammation is crucial for the induction of hepatocarcinogenesis. They even argued that the inhibition of inflammatory pathways might be a promising approach for the HCC prevention and therapy. They proposed the drug sorafenib as a candidate for such treatment but warned that promising experiments were only performed in mouse HCC models, which does not yet permit us to have it tested on humans. In contrast to Yu et al. (2018) and Dapito et al. (2012), on the basis of several highly sophisticated animal models, came to the conclusion that there is no significant contribution of intestinal microbiota and TLR4 to liver cancer induction but it is valid for the tumor promotion, mediating HCC proliferation, secretion of growth factors such as liver mitogen epiregulin and prevention of apoptosis. Nevertheless, the majority of the authors agreed that gut microflora derived from bacterial products. Some endogenous substances such as high mobility group B1 (HMGB1), free fatty acids collectively called DAMPs, in concert with TLRs, in the course of alcoholic and nonalcoholic liver disease, contribute to the hepatocarcinogenesis (Hernandez et al. 2019; Petrasek et al. 2013; Roh and Seki 2013).

## Other Liver Diseases

**Nonalcoholic steatohepatitis (NASH)** formation and development has been found to be TLR9 pathway activation dependent. Plasma from individuals with NASH contains high amounts of highly oxidized mitochondrial DNA, mostly in microparticles from hepatocytes. This DNA, relatively CpG rich, is able to activate TLR9. Mitochondrial DNA acts as a ligand of TLR9, which results in the stimulation of proinflammatory cytokines in hepatic microenvironment. Total body TLR9 deletion results in the disappearance of NASH in high fat diet (HFD) fed mice. TLR9 antagonist such as IRS954 prevents NASH formation in HFD fed mice (Garcia-Martinez et al. 2016).

**Autoimmunity**—there are reports in the literature that endogenous ligands such as nucleic acids RNA and DNA derived either from microorganisms or of mammalian origin may activate TLRs under certain conditions and initiate a typical autoimmune process such as systemic lupus erythematosus (Barrat et al. 2005; Hurst and von Landenberg 2008). Chi et al (2018) have shown in mouse models that stronger/long-lasting liver inflammation resulting from virus (adenovirus) or chemicals (CCl_4_) may lead to autoimmune hepatitis by releasing the ligands for TLR2/TLR4. Upregulated cytokines and chemokines in the liver activate STAT3 and STAT4 in hepatic CD4^+^CD25^+^ Treg cells suppressing FOXp3 gene expression resulting in the reduction of suppressive activity of Tregs.

**Malaria** parasites infestation—there is a growing opinion that PRRs, mainly TLRs recognize *Plasmodium* components and shape innate, albeit inadequate immune response (Kalantari 2018). The group of Japanese authors (Ernest et al. 2018) reported that the TLR2 agonist (PEG-Pam2Cys) dramatically reduces the quantity of rodent malaria parasite *Plasmodium yoelli* that grows in mouse liver. The drug was also able to clear parasites from the liver after establishment of infection.

**Gut–liver axis**—bile acid metabolism connects the liver with the intestine. Dysregulation of bile acids leads to intestinal dysbiosis that promotes pathogenic microorganisms entrance in the liver via portal vein and subsequent hepatic inflammation. Dysbiosis is also due to faulty bidirectional liver-microbiome crosstalk (Adolph et al. 2018). It triggers NLRs and the formation of inflammasomes. NLRP3 inflammasome induces proinflammatory cytokine IL-1β that aggravates inflammatory hepatic damage and affects the epithelial integrity of cholangiocytes. Bile acids are able to activate NLRP3 inflammasome in macrophages. In contrast, the NLRP6 one maintains microbial balance and antimicrobial peptide synthesis as well as mucus secretion by goblet cells (Wang et al. 2019).

**Liver transplantation**—in liver transplant recipients, the damage of cells during ischemia–reperfusion injury poses a significant clinical problem. Sosa et al. (2020) found TLR4/7/9 and NOD2 expression of portal blood may be used as a marker of cell damage for prediction and/or therapeutic intervention in orthotopic liver transplant patients before transplantation**.**

## Concluding Remarks

Pattern recognition receptors are still not fully appreciated by clinical hepatologists as an important factor in the management of liver diseases. The data provided in this article suggest that the complex nature of their function may hamper direct transfer of this knowledge to clinics. Nevertheless, the growing number of PRR synthetic ligands, showing therapeutic effect in various liver pathologies appears to be a promising agent for future liver therapies.

## Abbreviations

PRR: Pattern recognition receptor
RIG-1: Retinoic acid-inducible gene 1
TLR: Toll-like receptor
PAMP: Pathogen-associated molecular pattern
DAMP: Danger-associated molecular pattern
NF-κB: Nuclear factor kappa (light-chain-enhancer of activated B cells)
AP: Activator protein
IRF: Interferon regulatory factor
MHC: Major histocompatibility complex
HSC: Hepatic stellate cell
NLR: NOD-like receptor
TRIF: TIR-domain-containing adapter-inducing interferon-β
CpG-ODN: CpG oligodeoxynucleotide
pDC: Plasmacytoid dendritic cell
PBMC: Peripheral blood mononuclear cell
NK: Natural killer cell
Treg: Regulatory T cell
Th17: T helper 17 cell
HCC: Hepatocellular carcinoma
MDA5: Melanoma differentiation-associated protein 5
IPS-1: Interferon-beta promoter stimulator 1
SNP: Single nucleotide polymorphism
NOD2: Nucleotide-binding oligomerization domain containing 2
MD2: Myeloid differentiation protein 2
Tak1Hep: TGFβ-activated kinase 1
